# Perceived Help and Support for Sex as Self-Injury: A Qualitative Study of a Swedish Sample

**DOI:** 10.1007/s10508-022-02437-x

**Published:** 2022-10-19

**Authors:** Cecilia Fredlund, Linda S. Jonsson

**Affiliations:** 1 Department of Psychiatry in Linköping and Department of Biomedical and Clinical Sciences, Linköping University, 581 83 Linköping, Sweden; 2 Department of Social Sciences, Marie Cederschiöld University, Stockholm, Sweden

**Keywords:** Nonsuicidal self-injury, Indirect self-injury, Sexual abuse, Sex as self-injury, PTSD

## Abstract

Earlier research has found that sexual acts could be used as a means of self-injury, with comparable functions to nonsuicidal self-injury (NSSI) such as cutting or burning the skin. However, no previous study has investigated the experience of help and support in relation to sex as a means of self-injury (SASI), which this study aims to investigate. The study was based on an anonymous open-ended questionnaire published from December 2016 to April 2017 on the websites of NGOs working with help and support for women and youths in Sweden. In total, 197 participants (mostly women, mean age 27.9 years, range 15–64 years) with self-reported experiences of SASI were included in the study. Three main themes were found concerning experiences of help and support for SASI. The need for: (1) Framing the behavior of SASI, to find a word for SASI—to know it exists, to get questions and information about SASI and its function; (2) Flexible, respectful, and professional help and support from an early age, to be listened to and confirmed in one’s experience of SASI; and (3) Help with underlying reasons to exit SASI such as finding one’s own value and boundaries through conventional therapy, through life itself, or through therapy for underlying issues such as earlier traumatic events, PTSD, dissociation, or anxiety. In conclusion, similar interventions could be helpful for SASI as for NSSI, irrespective of the topographical differences between the behaviors, but the risk of victimization and traumatization must also be addressed in SASI.

## Introduction

In recent years, research has found that despite the topographical differences, sexual acts could be used as a means of self-injury, with comparable functions to nonsuicidal self-injury (NSSI) such as cutting or burning the skin. However, no previous study has investigated experiences of help and support in relation to the phenomenon described in the literature as sex as self-injury (SASI).

### What Is Sex as Self-Injury?

Nonsuicidal self-injury (NSSI) was included as a suggested diagnosis in need of more research in the fifth edition of the *Diagnostic and Statistical Manual of Mental Disorders* (DSM 5). It was defined as self-inflicted damage to the surface of the body without suicidal intention, with underlying motives of relieving negative feelings, resolving interpersonal difficulties, or inducing positive feelings (American Psychiatric Association, 2013), and it often refers to behaviors such as cutting, burning, or striking the skin. NSSI can be considered a direct form of self-injurious behavior, while abusive relationships, substance use, eating disorders, and risky or reckless behaviors can be considered indirect forms of self-injury (Nock, 2010; St. Germain & Hooley, 2012). However, the co-occurrence of indirect and direct forms of self-injury is common, and the behaviors can overlap regarding function and intention to cause direct injury to oneself. This might indicate that direct and indirect forms of self-injury are related behaviors that could be on a psychopathological continuum (D’Agostino et al., 2020; Fox et al., 2019; Jonsson et al., 2019). Emotional regulation and interpersonal functions are the most reported functions in relation to self-injury (Cipriano et al., 2017; Taylor et al., 2018).

The use of sex as a means of self-injury (SASI) has been described in studies in Swedish context in recent years. SASI could share the same function as NSSI such as cutting or burning the skin, and the two behaviors could even replace each other (Fredlund et al., 2020; Jonsson et al., 2019). Both NSSI and SASI have been found to function as a means of emotion regulation, for example “to relieve feeling numb or empty,” “to stop bad feelings,” or “to punish oneself.” However, social functions such as “to try to get a reaction from someone, even if it is a negative reaction” and “to get attention” were more commonly reported for SASI compared to NSSI (Jonsson et al., 2019). This has also been described in qualitative research. SASI can be used to regulate negative emotions such as depression, anxiety, numbing, dissociation, poor self-esteem, self-hatred, or reexperiences of earlier traumatic events. SASI could function as means to get positive confirmation, to be seen and validated, or to feel good at doing something (sex). It could also function in means to get negative confirmation of a poor self-image and self-contempt that was confirmed in sexual situations by being beaten or mistreated (Fredlund et al., 2020). Even though SASI lacks a common definition, it has been described as sexual situations that can include both psychological and physical harm that is sought as a means of self-injury. The manifestations of SASI could be diverse, such as having sex with a person without any attraction or desire, or letting the other person do whatever he wanted with the body without feeling any desire. SASI could include behaviors such as having sex despite not feeling attraction or desire, having new sexual partners every evening, contacting older men through the Internet for whom there was no attraction or desire, exposing oneself to sexual or physical violence in sexual situations, or repeating experiences of earlier sexual abuse by causing injury to one’s own genitals (Fredlund et al., 2020). Some have even described selling sex as a means of self-injury (Fredlund et al., 2018, 2020; Jonsson et al., 2015), although other reasons are more commonly reported in relation to selling sex among youths (Fredlund et al., 2018). SASI has been found to often start during adolescence (Fredlund et al., 2020). In a study including almost 6000 students from the third year of Swedish high school (mean age 18 years), 3.2% of girls and 0.8% of boys reported having used sex to intentionally hurt themselves on at least one occasion (Fredlund et al., 2017). In the same study, 11% of the adolescents with SASI also reported having sold sex on at least one occasion. In a pilot study of a US collage sample, 12% reported to have used sexual activities to self-injure (Mellin & Young, 2022). SASI could lead to a vicious circle with more and more self-destructive and dangerous sexual situations, including an increasing amount of violence and risk of victimization, which could be hard to break without help and support (Fredlund et al., 2020).

According to a review by Bresin (2020), behaviors such as NSSI, aggression, substance use, binge eating, and risky sexual behaviors are all examples of dysregulated behaviors with short-term benefits and long-term distress and impairment that can be hard to control. These behaviors can result in harm to the patient that can be explained by similar ethology and helped by similar interventions (Bresin, 2020). Self-injurious behaviors are related to a variety of psychiatric conditions such as personality disorders, anxiety disorders, depression, posttraumatic stress disorders, dissociation, substance use disorder, and suicidal thoughts and behaviors (Cipriano et al., 2017; Forbes et al., 2019; Kiekens et al., 2018a, 2018b), and strong associations have been found with earlier traumatic events, especially sexual abuse (Cipriano et al., 2017).

### Sex as Self-Injury and Sexual Abuse

SASI has been closely associated with experiences of earlier sexual abuse (Fredlund et al., 2017, 2020; Zetterqvist et al., 2018). Risk factors for SASI in comparison to NSSI were experiences of earlier sexual abuse, trauma symptoms of posttraumatic stress and dissociation, and a higher number of sexual partners (Zetterqvist et al., 2018). Earlier sexual abuse has been described as one reason for SASI to start, since SASI can give a feeling of control of the situation; it can be a way to regulate negative emotions or a way to punish oneself with regard to self-hatred after sexual abuse (Fredlund et al., 2020). Finkelhor and Brown (1985) described the long-lasting traumatic effects that can be seen after sexual violence in the Traumagenic Dynamics model. This model included the four traumagenic dynamics—traumatic sexualization, betrayal, stigmatization, and powerlessness—that are identified as the core psychological injuries inflicted by child sexual abuse (CSA), and are described as influencing the person’s self-concept, emotional state, and sexual relationships. Traumatic sexualization can explain sexual preoccupation, compulsive sexual behavior, and sexual aggression after CSA. It can also lead to negative attitudes toward sexuality and the body, sexual dysfunctions, and aversion to sex as an adult. Stigmatization is common after CSA, and is closely associated with shame, guilt, and a feeling of being different, which can lead to self-destructive behaviors including drug and alcohol abuse, criminal activity, prostitution, and suicide attempts. The betrayal in CSA may lead to a great need to regain trust and security, which in turn can result in impaired judgment about the trustworthiness of other people and a desperate search for relationships. It can also result in distrust, leading to isolation and aversion to intimate relationships. The dynamic of powerlessness may lead to the need to take back control, which can result in reenacting the experience of abuse but also a desire to dominate others (Finkelhor & Browne, 1985). These dynamics might be helpful when understanding SASI and the implications of interventions.

The association between earlier sexual abuse and later sexual risk-taking behaviors is well described in the literature, including increased risk of a greater numbers of sexual partners, a higher frequency of sexually transmitted infections, teenage pregnancy, selling sex, and younger age of sexual debut (Abajobir et al., 2017; Hailes et al., 2019; Lalor & McElvaney, 2010; Messman-More & Long, 2003; Scoglio et al., 2021; Senn & Carey, 2010; Steel & Herlitz, 2005). Child sexual abuse has been associated with compulsive sexual behaviors including sexual impulsivity, hypersexuality, out-of-control sexual behavior, problematic sexual behavior, and sexual addiction (Slavin et al., 2020). Emotional dysregulation has been found to be an important factor for sexual risk-taking behaviors in relation to posttraumatic stress disorder (Weiss et al., 2019), and dysregulated emotional control has been found to explain the indirect relationship between self-injurious behavior and sexual risk-taking behavior (Marraccini et al., 2019). As described earlier, SASI can be forced by the function of the behavior and can include sexual risk-taking, including sex with poorly known partners and sexual situations including physical and sexual violence. Hence, this can increase the risk of further victimization and might be one reason for the association between earlier sexual abuse and later sexual risk-taking behaviors and revictimization.

### Sex as Self-injury, and Help and Support

A recent interview study investigating the connections between child sexual abuse, disclosure, and self-injurious thoughts and behaviors found that self-injury was a way to cope with the experience of abuse, end victimization, and deal with the self-hatred and loneliness associated with the sexual abuse. Negative disclosure experiences can result in increased self-injury (Collin-Vézina et al., 2021). To our knowledge, experiences of help and support regarding SASI have not been studied previously. However, a systematic review has been carried out concerning experiences of professional care and support among persons with NSSI. Important factors described were healthcare professionals who were competent, supportive, and prepared to listen, and who provided satisfactory treatment. The person needed to be respected, seen, and heard, which gave them confidence and helped them to make progress. Staff needed to be understanding, non-judgmental and encouraging, proactive, and competent. Continuity was also important, and therapy was vital. Negative experiences from health care that discourage people from seeking help might accelerate self-injury (Lindgren et al., 2018). Recovery from NSSI has been described as a process that might not have a definitive endpoint, but building up resilience by developing other skills that can replace NSSI has been described as helpful, as has help with underlying mental illness (Lewis et al., 2019).

Barriers to seeking health care and disclosure have been studied for sexual violence, such as in sexual exploitation, and can include stigma, feelings of shame, and worries about negative reception from professionals (Barnert et al., 2017). Symptoms of PTSD are the most common reason for seeking health care after sexual abuse (Amstadter et al., 2010), but other psychiatric problems such as drug and alcohol abuse, anxiety, depression, and pain are also common reasons for seeking health care in relation to sexual abuse (Rajan et al., 2017). Help and support for the psychological effects of sexual abuse are often delayed; in a Swedish study, the average time before seeking help was found to be 15.9 years for victims of sexual abuse (Rajan et al., 2021).

In conclusion, the phenomenon of SASI is a relatively new field of research that has been studied in Swedish contexts that share common functions with NSSI. SASI is closely connected to earlier experiences of sexual abuse and is a behavior that might increase the risk of sexually transmitted infections, unwanted pregnancies, and further traumatization and victimization. In the literature, there are studies investigating help and support in relation to sexual violence and NSSI, but there is currently a lack of knowledge concerning experiences of help and support regarding SASI. It is important to understand how help and support can be modeled and experienced in relation to SASI, and whether similar interventions could be used for SASI as for NSSI or sexual violence.

### Aim of the Study

The aim of the study was to increase the understanding of perceived help and support for persons with experience of SASI. There were three research questions, investigating how persons with SASI experience help and support:

- What kind of help and support is perceived as helpful versus not helpful?
- What is helpful in the exit process?
- How can sufficient professional support be structured regarding SASI?

## Method

### Participants

The study used a qualitative design and was based on an open-ended questionnaire. In total, 197 participants with self-reported experience of SASI were included in the study. Data were analyzed following the six steps for inductive thematic analysis described by Braun and Clarke (2006).

Participants were recruited to the study during the period December 2016–April 2017 via advertisements published on the websites of non-governmental organizations (NGOs) such as women’s shelters or NGOs supporting women’s and young people’s sexual or mental health. The study was advertised as follows: “Do you have experience of sex as self-injury and are over 15 years of age? Do you want to participate in an anonymous questionnaire-based study in order to increase understanding of SASI and improve help and support? Click on this link.” In total, 37 NGOs from all parts of Sweden agreed to publish the link to the study. The NGOs were chosen in view of earlier findings indicating that adverse events are common among persons with experience of SASI, and the study group was therefore expected to visit these kinds of websites (Fredlund et al., 2017). To be included in the study, participants had to have self-reported experience of SASI and be 15 years of age or older, since according to the 2003 Swedish Ethical Review Act, consent is not required from parents when participants are 15 years of age or older. The questionnaire was answered anonymously in view of the sensitive topic, and in the introduction to the questionnaire a definition of SASI was presented as having “Repeatedly sought sexual situations that have caused you physical and/or mental harm and that have affected you in your life.” To collect the answers, web-based survey software—Artologik Survey and Report, procured by Linköping University (Artologik, 2021)—was used.

In total, 199 participants (190 women, four men, four people with non-binary gender identification, and one person with unknown gender) answered the questionnaire, but 197 were included in the analysis since two women did not answer the questions concerning help and support. The age range for the participants was 15–64 years, with a mean age of 27.9 years (SD = 9.3). SASI had started during adolescence for most of the participants (85.4%).

### Measure

The questionnaire was written in Swedish and included 12 open-ended questions that were specifically designed for the study. The questionnaire was tested in a pilot study including five informants in October 2016. The pilot study participants requested more precise follow-up questions, and the questionnaire was amended accordingly. The questionnaire included open-ended questions concerning coping strategies for negative feelings, experiences of sex as self-injury, and the reasons why SASI started, continued, and stopped, and can be read in full (see Fredlund, 2019). The questions concerning experiences of help and support and reasons for exiting SASI were formulated as follows: (1) If you have stopped, what made you stop? (2) What experiences do you have of help and support when you had sex as self-injury? (3) What did you want regarding help, support, and treatment from health care or other organizations working with help and support when you had sex as self-injury?

### Data Analysis

Thematic analysis followed the six steps described by Braun and Clarke (2006). The data set was first read and re-read several times to become familiar with the data by the two authors. Initial codes were generated throughout the data set and themes were identified by sorting and re-reading the initial codes and the data connected to the codes. The themes were checked in relation to the coded extracts and the total data set, and were processed again to be redefined and clarified into more distinct themes with regard to internal and external homogeneity. Themes were strengthened using quotations in the written text and presented in a coherent pattern. The NVivo 12 Pro software was used as support for the analysis and to keep track of the citations used. The coding and thematization of the total data set were carried out by the first author (CF), and triangulation was conducted by the last author (LJ) by both researchers coding parts of the data separately, enabling discussion and comparisons of coding categories and coherence. A coding protocol was used to keep track of decisions and to map the path toward the themes. Decision trails were kept ensuring the accuracy of the coding during the process. To gain validity or trustworthiness in relation to the coding, different strategies have been used. The first author started coding and developed the code book. After that the two authors independently coded part of the data and the results were compared. Thereafter the codebook was adjusted, and some codes were merged, some erased and some added. During the further analysis and thematization, a throughout discussion took place and the themes and subthemes were named and renamed until both authors were satisfied with the results. Quotes were picked out and the original data file revisited before everything was finalized.

The length of the answers varied considerably from one participant to another, consisting of between a few words per question and half a page. All the included informants contributed at least one coded extract to the analysis. All answers were written in Swedish, and the quotations were translated into English for the article with the support of professional translators in dialogue with the researchers.

## Results

Three main themes were identified in relation to experiences of help and support for SASI in the study: (1) Framing the behavior of SASI, (2) Flexible, respectful, and professional help and support from an early age, and (3) Help with underlying reasons to exit SASI—see Table 1. An additional important finding, not related to the study question, was that 137 out of 197 participants reported having received no or unsatisfactory help and support regarding SASI and has to be investigated in further research.

**Table 1:** Main themes and subthemes for perceived help and support with regard to sex as self-injury

*Framing the behavior of SASI*
To find a word for SASI—to know it exists
To be asked about SASI
To get knowledge and information about SASI
*Flexible, respectful, and professional help and support from an early age*
Respectful and professional contact with health care
To receive help early on, during childhood
The need for flexibility in relation to help and support
*Help with underlying reasons to exit SASI*
Finding one’s own value and boundaries
Life events as turning points
Conventional therapy and help with underlying reasons

### Framing the Behavior of Sex as Self-Injury

*To find a word for SASI—to know it exists* was the most fundamental need in order to receive help and support for SASI. Because without a word for SASI, it was hard to frame the behavior with regard to the function, it was hard to ask for help and support, and it was hard to receive proper help and support. The informants described how it could take time to understand that the sexual act was destructive and that it was a form of self-injury. A 22-year-old woman (Ax150) described her experiences as follows:

“It became like a compulsion and an addiction. First because I did not understand that it was destructive, and later because I could not stop. I tried to sign a contract with myself. Use all the strategies I used to stop cutting myself. But I could not resist. The shame also contributed. Although I have been in therapy for the last few years, I did not dare to talk about this. I was so ashamed. I was sure my therapist would hate me, think I was disgusting, and throw me out if I told her. […] I could not ask for help.”

The behavior included severe feelings of shame and guilt, which made the informants unwilling to talk about the behavior, but finding a word for SASI was helpful in terms of reducing the shame and guilt. A 15-year-old girl (ax177) wrote:

“If I had sought help, I would have wanted to meet someone who explained that sex as self-injury was a thing. I didn’t know why I did what I did, I just wanted it to stop while I wanted it to continue, while I wanted it to hurt more. If someone had explained to me why I did it, I probably wouldn’t have had as much guilt.”

*To be asked about SASI* was frequently described as desirable by the informants, especially that the question should be asked within a healthcare context and in relation to other kinds of self-injuries. This desire was expressed by a 28-year-old woman (Ax110) as follows: “First of all, I wish someone had asked me/talked to me about this, because I had several other self-injurious behaviors. I needed help to understand that this could also be self-injury.”

Informants experienced that not being asked about SASI increased their shame about the behavior, but it sometimes took time to be ready to talk about the behavior. Informants described that the first time the question was asked, the person might not be ready to talk about the behavior, but the question concerning SASI needed to be asked repeatedly. A 27-year-old woman (Ax84) wrote: “I wish someone had just asked at all. I also wish I had been asked several times. I would have liked to talk to someone about it, to have turned to someone who understands what it’s about.”

Informants even believed that just being asked about SASI by healthcare workers and putting SASI into words could be helpful for leaving the behavior. A 33-year-old woman (Ax47) wrote about her desire for help and support:

“To help me understand that I had an unhealthy relationship with sex. I had contact with psychiatric care throughout this whole period. The question could have been asked. Then it might not have had to last for so many years.”

Informants had experiences of long-term contact with psychologists, psychiatric clinics, or other parts of the healthcare system, but had never been asked questions related to SASI. Informants described that they had tried to talk about SASI with their caregivers but had not been listened to or understood. A 35-year-old woman (Ax46) wrote:

“Mentioned it to several different therapists over the years, and was just met with silence and it was not something that we ever talked about again. Therefore, I did not dare to tell my last therapist until after two years when I had stopped doing it myself. Experienced peace and security, and was able to let it go for good after that.”

*To get knowledge and information about SASI* and the function of the behavior were described as important to be able to leave the behavior, but informants found it hard to find a professional care unit with knowledge regarding SASI. Informants even described experiences of encountering ignorance within health care regarding the function of SASI. This was described by a 28-year-old woman (Ax63):

“I wish the psychologist I went to for several years had understood and listened to me, heard the warning signs and talked to me about this. Everything was just to be medicated away, but it led to me living even more destructively. I wish they had talked to me about this, to turn bad feelings, panic, and traumatic stress toward oneself, that self-injurious behavior could take different forms and so on.”

However, other areas of society also needed to address SASI to break the taboo and shame. A 30-year-old woman (Ax170) wrote: “Would never dare to talk about it. The stigma and taboo are too great. Society needs to talk about it. That it exists.”

### Flexible, Respectful, and Professional Help and Support from an Early Age

*Respectful and professional contact with health care* was described as important for the informants. To be confirmed and seen was the most crucial aspect of contact with a person with experience of SASI—not only within health care and with therapists, but also in other parts of society. The informants described that they wanted to be treated with respect and understanding. They wanted to be listened to, to be seen, and confirmed in their experiences. They wanted to be taken seriously, and for the person to believe in them. A 24-year-old woman (Ax111) wrote: “If I had sought help, I would have liked to be met with understanding and someone who listened.” Another 33-year-old woman (Ax156) described it as follows:

“I had wanted to talk to someone who was more understanding and did not look at me as if I was crazy and awful. Someone who could help me to sort through my feelings and not just make me feel awful.”

Shame and self-blame were common in relation to SASI, and were important reasons for not talking about SASI and hence not getting help. However, experiences of caregivers blaming the person and giving her more shame and guilt were also described. A 27-year-old woman (Ax122) described her experiences as follows*:*

“I have not received help. If I spoke about it in situations that involved seeking help, people looked at me strangely and seemed to think I should blame myself. Including at the women’s shelter. They do not think that the men did anything wrong, even though there was very serious and dangerous violence at times, such as strangulation. The attitude I usually encounter is that I should blame myself for getting myself into it. Including within psychiatry. But the most common thing in psychiatry is that they just do not understand that it is a problem for me, even though I have often tried to make them understand. They think I have the right to do as I please. And then I stop talking about it.”

Caregivers needed to remain professional and deal with their own feelings and reactions in relation to what were sometimes very traumatic life stories from persons with SASI. A 55-year-old woman (Ax65) wrote: “My counselors and psychologists cried when I talked about my life story, and I never felt any support.”

*To receive help early on, during childhood* was a recurrent theme among the informants in relation to SASI. Most of the informants had started to use SASI during adolescence, while aged 12–19, and the informants found it important to get help at this age. A 27-year-old woman (Ax75) expressed this desire as follows: “That the staff at the youth clinic would have seen my patterns and dared to ask. I was there more than once a week for various tests.” Another 27-year-old woman (Ax163) wrote:

“I wish someone had noticed me as a child and could have stopped it then. I had early sexually risky behavior (and other things as well) that was visible. Nevertheless, no report was made to social services. If someone had dared to pay attention to me and protect me, it might never have had to be that way. Then maybe I would have felt well enough not to be attracted by destructive behavior. I have been raped so many times that I cannot keep count. I wish it could have been stopped before it happened. Back when I was a child […].”

Informants said that it was important to ask about SASI not only within healthcare contexts, such as child and adolescent psychiatry, and youth clinics working with sexual health, but also within school health, social services, and other services working with young people. It was suggested that sexual relationships, consent, and SASI should be discussed in school lessons. A 19-year-old woman (Ax115) wrote: “First of all, shame must be removed. You are shamed for allowing yourself to be exploited. Schools need to talk on a broader and deeper level about sex, sex as self-injury, consent, etc.”

*The need for flexibility in help and support* for SASI was important. This involved issues such as the need to offer female therapists (since dealing with men could trigger memories of earlier traumatic events), the need for anonymity (such as receiving online support), and the need for continuity and not being sent from person to person to get a feeling of security. A 28-year-old woman (Ax180) wrote:

“I have tried to seek help many times, but I have only been sent to person after person. I’ve had to tell my story easily 30 times to completely different people, and then I have not received any help anyway or then they have realized that they do not have the skills to help me, so they send me on once again to someone else […].”

Respondents described a need for accessible care with drop-in appointments, which might not be limited to daytime hours, such as internet forums and support. Knowing that there are others with SASI was also found to be helpful, and was suggested to be achieved via group therapy, help organizations, or online forums. A 39-year-old woman (Ax113) wrote:

“I told the healthcare workers that I had been sexually exploited, sexually assaulted, and raped, but said that I did not want to go into detail and did not want to tell them more. What I wished for was to have been given advice about a forum where you can be anonymous and read how others have handled the situation, and maybe then I would had dared to start talking about it.”

A 34-year-old man (Ax149) wrote:

“The years I devoted myself to this I had no support at all. I sometimes looked for information online. It gave me the impression that there is not much help available. After I sought care that consisted of conventional group therapy, it was very valuable to realize that I was not alone in this. To share one’s own experiences with others who can relate to them has been the key to healthy sexuality.”

### Help with Underlying Reasons to Exit Sex as Self-Injury

*Finding one’s own value and boundaries* was an important factor for leaving SASI. Poor self-esteem, self-hatred, and self-contempt were common reasons for SASI. The informants asked for help to find their own value and boundaries to be able to leave SASI, as well as help with other underlying reasons for SASI. A 25-year-old woman (Ax117) wrote: “Above all, I was helped to realize my own value as a person and an individual. I was helped to put my feelings into words and to stay in the anxiety instead of numbing the pain inside.” A 27-year-old woman (Ax101) wrote:

“I had wanted to be supported by the counselor. For her to tell me it was not my fault, that only I have the right to my own body, and for her to try to help me to report my perpetrator. Maybe I would never have started using sex as self-injurious behavior if I had received that help then. I had wanted help with setting boundaries, I couldn’t do it by myself.”

Finding their own value and boundaries could be achieved in different ways, such as through therapy, but another common theme was that the person had entered a stable relationship or met a partner who helped her to find her value and boundaries. A 25-year-old woman (Ax130) wrote*:* “I met a partner who appreciates me and makes me look at myself as an individual. Someone who deserves to decide for myself about my sexuality. As well as many hours of therapy.”

*Life events as turning points* were often described as occasions when the person realized that she must stop or find a way out of SASI. This could involve meeting a stable partner, getting into therapy, having children, simply growing older, improving her mental health, or her secret life of SASI being revealed. A 28-year-old woman (Ax180) wrote about her reasons for stopping using SASI as follows:

“That I met my current boyfriend and partner. He questioned my approaches, and when he saw that I did not want to. He has followed me and paid for therapy so I will feel better. He has been a huge support for me, and would never have sex with me if he knew I didn’t want to. He himself has stopped several times when he noticed that I was not committed, and encouraged me to say no when I don’t want to.”

A 27-year-old woman (Ax101) wrote: “I found the strength to quit when I got older and made better friends. I felt better myself and began to understand my own value better. I think it was a lot about getting older.”

A 25-year-old woman (Ax108) wrote:

“I became pregnant and felt that my child did not deserve that treatment. I did not think she deserved to be hurt, it was just me who would be hurt. My body was no longer just mine, so I couldn’t subject it to bad treatment.”

Another turning point could be “reaching the bottom” of very poor mental health, in the form of either a suicide attempt or a situation where the person was raped or badly abused during SASI. A 20-year-old woman (Ax1) wrote: “After trying to kill myself, I had to see a psychologist. That was where things turned around. I found peace and understanding for myself on a whole new level.” A 24-year-old woman (Ax111) wrote: “After I was raped, it was as if I had had enough and decided that now it should be enough.”

Avoiding situations that could trigger SASI and instead being in environments that respected the individual’s value and boundaries were also described as turning points for leaving SASI. This could involve not having sex with men and having sex with women instead, not drinking alcohol, making new friends with different views about sex, avoiding the internet, or stopping having sex entirely. A 26-year-old woman (Ax70) described how SASI stopped for her:

“Better living conditions. I got a job that I managed to do well, and became popular. A little better mental state that meant that even though I can’t feel it emotionally, I can admit intellectually that I deserve better. Because I can see that, I avoid all situations where I can fall back, and I have realized that I have to isolate myself to some extent until I can really value and respect myself. I cannot trust myself to make wise decisions in vulnerable situations, and therefore I must not be in such situations.”

A 23-year-old woman (Ax197) wrote: “I fell in love with a girl when I was 16. Since then, I have gradually stopped. Partly because I stopped having sex, but also because I have started being with girls instead of boys.”

Informants found it important to be seen and helped by family and friends. A 28-year-old woman (Ax33) wrote: “A close friend realized what was going on and made me understand what I was doing, which helped me stop. But I also got a steady partner.”

*Conventional therapy and help with underlying reasons* for SASI were the most important kind of help and support informants had received or wanted to receive from health care. Different forms of therapy were described as helpful, such as trauma therapy, therapy for self-injury, or simply conventional therapy. Getting therapeutic help and help with the underlying causes—such as traumatic experiences, posttraumatic stress syndrome, poor self-esteem, dissociation, anxiety, sleeping problems, need for confirmation, emotional regulation, and self-injury—were found to be desirable and helpful. A 40-year-old woman (Ax119) wrote: “I have tried to cut down on it because I have realized that it injures me. But it is hard!! Because I have to find another channel for anxiety relief that does not injure me.”

Earlier traumatic events were frequently described as underlying reasons leading to the use of SASI. A 17-year-old girl (Ax87) wrote: “I would have liked more competent psychologists and doctors who had been educated that young people who have been raped often do this to themselves.” A 20-year-old woman (Ax83) wrote: “No, I have not stopped. But I think if I can start and complete the PTSD therapy I have been offered, I can process everything enough to find myself again.”

Some had experiences of dialectical behavior therapy (DBT), which is often used for borderline personality disorder, suicidal ideation, and self-injury. Some had found it helpful, but others had never been able to talk about SASI in therapy. A 24-year-old woman (Ax30) wrote about her experiences of help and support: “None at all. I have been in DBT and had a psychologist for other reasons, but the topic never came up in the conversations and I didn’t bring it up myself.” A 33-year-old woman (Ax40) wrote about her desire for help:

“Some tool to get over the total feeling of worthlessness. I got help with cutting, my shitty relationship with food and drink, but no one ever mentioned sex. Neither did I, in fact, but I was not fully aware that what I was doing was self-injury. I have understood that afterwards. Caregivers need to be aware of the existence of the behavior and need to get their patients to talk about it. Especially in DBT, as it is a fairly common behavior among borderline persons.”

## Discussion

SASI is a relatively new phenomenon that is still sparsely described in the literature. The aim of this study was to investigate experiences of help and support among informants with SASI, since no previous study has been found on the subject. The most important findings regarding experiences of help and support for SASI were: (1) The need to frame the behavior of SASI, (2) Flexible, respectful, and professional help and support from an early age, and (3) Help with underlying reasons to exit SASI. These findings will be discussed further below.

First, the informants found it important to get a word for the behavior to be able to frame what they had experienced. This was necessary to get help and support, but also to be able to leave the behavior. Questions, knowledge, and information were found to be helpful for quitting the behavior, but a lack of questions, information, and knowledge increased the stigma, shame, and self-blame. Some informants were unable to put their experiences into words because of shame and self-blame, which is why the questions needed to be asked repeatedly. SASI is a new and sensitive issue in the research field, since there is currently no common definition and the research is sparse and limited with regard to cultural contexts, hence the knowledge within society is limited. Questions may even be raised about whether it actually exists as a problem, since there are only a few studies framing the phenomenon as SASI. However, in Sweden, there is a debate related to the phenomenon where people, both professionals and victims, can relate to the concept and view it as helpful, and it has also been discussed in court cases. SASI could possibly be a cultural problem, since it has only been described in a Swedish context, but the description and functions of the behavior are most likely relevant to other nationalities as seen in a recent pilot study from the USA (Mellin & Young, 2022). Reflections could further be made on whether it could be harmful to define SASI as a self-injurious behavior, since it could be perceived as imposing guilt on a person with experience of sexual violence. According to this study, putting SASI into words was not experienced by the informants as imposing guilt, but rather increased the possibility of getting help and being able to leave the behavior. However, making the behavior invisible by not asking questions, not framing it as a self-injury, or not understanding the underlying reasons for the behavior increased the shame and guilt. On the other hand, when a new phenomenon such as SASI is described, it is also important to not stigmatize or pathologize normal sexual behaviors such as experiences of many sexual partners or norm-breaking sexual preferences that could have nothing to do with self-injury. The informants in this study reported a great lack of knowledge and information among healthcare and social workers, and requested greater competence concerning how SASI could be forced by the function of the behavior. In Sweden, there is an ongoing project to facilitate the screening of sexual risk-taking behaviors within the school health system and youth clinics, using an instrument as a tool for asking sensitive questions concerning sexual risk-taking behaviors (Hammarström et al., 2019). This might also be a way to ask questions about SASI. The informants highlighted the importance of asking about SASI within health care, especially in areas such as psychiatry, youth clinics, and clinics working with sexual health and self-injurious behaviors, but also in school health.

Second, earlier literature concerning help and support for NSSI has found that how the person is treated is very important (Lindgren et al., 2018), and validation is a key component of DBT that is commonly used for personality disorders with NSSI or suicidal behaviors (DeCou et al., 2019; Linehan, 1993). As has been found in studies concerning NSSI, informants with SASI described in this study that they wanted to be confirmed in their experiences, to be seen and treated with respect and understanding. The person needs to feel that she has been listened to and that the caregiver believes in her. Caregivers need to be non-judgmental and encouraging, proactive, and competent. Negative experiences from health care discourage people from seeking help, which might accelerate self-injury (Collin-Vézina et al., 2021; Lindgren et al., 2018).

Third, knowledge about NSSI has grown in recent decades, and NSSI was included as a suggested diagnosis in need of more research in the fifth edition of the *Diagnostic and Statistical Manual of Mental Disorders* (American Psychiatric Association, 2013). It was defined as injuries directed directly to the surface of the body, such as cutting, hitting, and burning the skin, without suicidal intention and with underlying motives of relieving negative feelings, resolving interpersonal difficulties, or inducing positive feelings. However, research in recent years has found that indirect forms of self-injury, such as aggression, substance use, binge eating, and risky sexual behaviors, are dysregulated behaviors that can share common functions with NSSI, with short-term benefits and long-term distress and impairment that can be hard to control (Bresin, 2020; D’Agostino et al., 2020; Fox et al., 2019; Jonsson et al., 2019). These kinds of behaviors can result in harm for the patient, which can be explained by similar ethology and helped by similar interventions (Bresin, 2020), as also described by the informants in this study. Comorbidity between indirect and direct forms of self-injury is common, and the behaviors can overlap with regard to function and intention of causing direct injury to oneself (D’Agostino et al., 2020; Fox et al., 2019; Jonsson et al., 2019). Earlier research found that SASI can share common functions with NSSI, and that these behaviors can even replace each other (Fredlund et al., 2020; Jonsson et al., 2015, 2019). This means that if the person stops cutting herself, she might continue with other forms of self-injury that could be even more destructive and dangerous with regard to physical and mental injury, such as SASI (Fredlund et al., 2020). It is therefore not enough to address one kind of self-injurious behavior in treatment; instead, the function of the behavior should be addressed, and the person should be helped to find tools to deal with all kinds of dysregulated behaviors such as NSSI, eating disorders, SASI, or substance abuse. If not, these behaviors might evolve into other self-destructive behaviors (Fredlund et al., 2020). This was also highlighted in this study by informants describing the importance of talking about SASI in treatments such as DBT and other therapies for self-injurious behaviors.

Fourth, the need for proper help and support in relation to SASI during adolescence was a theme identified in this study, since this was the age when SASI often started. Informants reported that it was hard to get help during adolescence, and that they found it important to highlight SASI within school health, social services, and other care units for adolescents. Another important issue regarding the organization of help and support was the need for anonymity and the need to meet others with the same experiences. In this respect, the internet was found to be an important forum for information and for meeting others with similar experiences, e.g., via chat forums. Internet-based treatment is a growing area within psychiatry, and the knowledge concerning interventions in self-injurious behavior is promising but still limited (Arshad et al., 2020). This form of intervention might be helpful for SASI.

Fifth, instead of just putting SASI into words, informants also found it important to get help with the underlying reasons for SASI. This includes treatment for earlier traumatization, including experiences of rape and physical violence, treatment for PTSD, anxiety, depression, emotional instability, and self-injury. Informants found it important to work on their own self-esteem and the ability to maintain their own boundaries. Conventional therapy was frequently described as helpful in this respect, as were support from friends and family, and not least meeting a stable partner. Lifechanging events were described as a point where the person got help or realized that she needed help and started to find her own value and boundaries. For some informants, life itself—due to getting older or having children—was sometimes enough to find the way out of a destructive pattern of self-injury including SASI. As seen in previous studies, SASI has been associated with earlier traumatization, not least sexual victimization, PTSD, dissociation, and poor mental health, and informants have also described SASI as increasing the risk of revictimization (Fredlund et al., 2017, 2020; Zetterqvist et al., 2018). This means that therapy focusing on PTSD and trauma might be an important part of helpful treatment for SASI, since self-injury might be just one way to cope with the experience of abuse, end victimization, and deal with the self-hatred and loneliness associated with the sexual abuse, as seen in previous studies (Collin-Vézina et al., 2021). According to this study, trauma treatment such as therapy for PTSD, dissociation, anxiety, and sexual violence, combined with therapy for emotional regulation, could be helpful in SASI.

Sixth, according to earlier research on SASI, experiences of sexual abuse before SASI are common, even though not all the informants had these experiences (Fredlund et al., 2017, 2020; Zetterqvist et al., 2018). SASI can be a way to take back control after sexual abuse, but it can also be a way to regulate negative emotions or a way to punish oneself with regard to self-hatred after the experience of sexual abuse (Fredlund et al., 2020). SASI can be understood as a sexualized behavior after sexual abuse that has been described as traumatic sexualization in earlier research (cf. Finkelhor & Browne, 1985), with the function of emotional regulation just like other kinds of self-injury (Fredlund et al., 2020; Jonsson et al., 2019). However, the relationship between SASI and sexual abuse is rather complex, since it has been described how SASI can also include sexual violence such as sex without inner desire or attraction as a way to punish oneself, physical and sexual violence, sexual exploitation through selling sex, exposing oneself to situations that involve a risk of victimization, and being exposed as a child (Fredlund et al., 2020). These are situations that should be described as sexual violence, and the person must of course be considered as a victim of violence, but to understand the function of SASI could be helpful for the understanding of underlying motives for returning to the violence repeatedly. More research is needed with regard to understanding SASI as traumatic sexualized behavior after sexual abuse (Finkelhor & Browne, 1985), or whether it could rather be described as an entity of sexual violence or as a self-injurious behavior alone. This is important not least in legal cases, since SASI could include extremely vulnerable situations that the person has been forced into due to poor mental health and a need for emotional regulation. When such cases come to court, the victim is often blamed for seeking risky sexual situations of her own accord. We suggest that the person should instead be seen as a victim of both sexual violence and poor mental health which leads to a more vulnerable position of exploitation and hence could be seen as more severe kind of victimization. This kind of exploitation should be seen as more severe and more resources for help and support might be needed since the poor mental health is forcing the vicious loop of the behavior (Fredlund et al., 2020).

### Strengths and Limitations of the Study

In this study, anonymous questionnaires were used instead of interviews, in view of the sensitive questions asked. This gave the study both strengths and limitations. First, the design made it possible to reach a large and diverse group of informants, including both those with ongoing SASI and those who had stopped using SASI. The informants were diverse in terms of age (15–64 years of age), but also in terms of their experiences of help and support. However, the design of the study made it impossible to ask further questions and to go into themes in greater depth. The answers were also shorter and less detailed than what we would expect in interview situations. On the other hand, the number of participants (197) who answered the questionnaire was high, suggesting that we have found the most important themes concerning experiences of help and support.

An important limitation concerning the concept of SASI is the limited number of studies and the lack of descriptions from other cultural contexts. This study was initiated in 2016–2017, but in recent years the concept of SASI has been more widely addressed within society in Sweden and SASI has also been included in the definition of self-injury used by the Swedish National Self-Injury Project (Nationella Självskadeprojektet, 2021). This means that the situation described by the informants—with regard to the way they were treated and the lack of information and knowledge about the behavior—might now be slightly different in Sweden today. More education has been provided concerning self-injury in recent years, but also concerning SASI within different forums such as conferences for school nurses, NGOs working with vulnerable young people and adults, healthcare staff, and midwives.

### Conclusions

In conclusion, this study aimed to investigate perceived help and support with regard to SASI, which to our knowledge has not been investigated previously. The study found that the most important experiences regarding help and support in relation to SASI were to get a word for SASI, to know it exists, and to get knowledge, information, and questions about the behavior. As with other kinds of self-injurious behaviors, the treatment needed to be flexible, respectful, and professional, and underlying reasons needed to be addressed. Conventional therapy or working with one’s own value and boundaries through life were helpful to be able to leave SASI. This means that similar interventions that have been found helpful for other kinds of self-injurious behaviors could also be helpful for SASI. However, in contrast to NSSI, SASI often includes another person, meaning that SASI could entail less control over the self-injury in the actual situation, and the person could be more vulnerable and at risk of victimization. More research is needed to understand the complex relationship between SASI and sexual violence, as well as studies of SASI in other cultural contexts, which is a limitation in the current field. It is hoped that the findings from this study will be helpful when developing help and support for SASI at healthcare units such as youth clinics, psychiatry, and school health, and in a further step prevent victimization and traumatization, which can be serious consequences of SASI.

## Data Availability

Not applicable due to ethical approval.

## Code Availability

Coding is available on request.
